# A simple and efficient fluorescent labeling method in *Staphylococcus aureus* for real-time tracking of invasive bacteria

**DOI:** 10.3389/fmicb.2023.1128638

**Published:** 2023-02-10

**Authors:** Fei Liu, Sijie Chen, Yingxin Zou, Yong Jiao, Ying Tang

**Affiliations:** ^1^Naval Medical Center, Naval Medical University, Shanghai, China; ^2^Department of Nursing, The 940th Hospital of Joint Logistic Support Force of People’s Liberation Army (PLA), Lanzhou, China

**Keywords:** *Staphylococcus aureus*, cyanine 5.5, heat shock, macrophages, bacterial labeling

## Abstract

Bacterial fluorescent labeling is a powerful tool for the diagnosis and treatment of bacterial infections. Here, we present a simple and efficient labeling strategy for *Staphylococcus aureus*. Intracellular labeling of bacteria was achieved by heat shock using Cyanine 5.5 (Cy5.5) near-infrared-I dyes in *S. aureus* (*Cy5.5@S. aureus*). Several key factors, such as Cy5.5 concentration and labeling time, were systematically evaluated. Further, the cytotoxicity of Cy5.5 and the stability of *Cy5.5@S. aureus* was evaluated by flow cytometry, inverted fluorescence microscopy, and transmission electron microscopy. In addition, *Cy5.5@S. aureus* were used to explore the phagocytic behavior of RAW264.7 macrophages. These results proved that *Cy5.5@S. aureus* had a uniform fluorescence intensity and high luminance; additionally, our method had no significant adverse effects on *S. aureus* compared to unlabeled *S. aureus* infections. Our method provides researchers with a useful option for analyzing the behavior of *S. aureus* as an infectious agent. This technique can be broadly applied to study host cell–bacteria interactions at the molecular level, and to *in vivo* tracing of bacterial infections.

## 1. Introduction

*Staphylococcus aureus* is one of the most common bacterial pathogens, causing an incalculable number of uncomplicated skin infections and hundreds of thousands to millions of serious invasive infections worldwide each year (Rasigade et al., 2014; Zhao et al., 2020). *Staphylococcus aureus* is the primary causative agent of pneumonia, other respiratory infections, surgical sites, artificial joint, cardiovascular infections, and intranasal bacteremia (Tong et al., 2015; Si et al., 2020). Owing to the widespread use of antibiotics in recent decades, *S. aureus* has rapidly developed multiple antibiotic resistances, thereby increasing the risk of fatal infections in immunocompromised patients (Willyard, 2017). Labeling and subsequent imaging/tracking of *S. aureus* will enable analysis of its dissemination, colonization, and induction of inflammation (Drevets and Elliott, 1995).

Bacterial fluorescent labeling technology is a powerful tool widely used in studies on bacterial detection (Mazrad et al., 2017; Geng et al., 2020), bacterial infection tracing (Welling et al., 2019; Chen et al., 2020; Feng et al., 2021), and antimicrobial therapy (McCollum et al., 2021). Fluorescent labeling facilitates the localization and tracing of target bacteria, allows the analysis of bacterial proliferation and dissemination, and helps our understanding of the interaction between bacteria and the immune system (Drevets and Elliott, 1995). Therefore, this labeling technology plays an important role in diagnosing and treating bacterial infections. Currently. A common labeling method for bacteria involves the use of cationic molecules that are electrostatically attracted to negatively charged cells (Yang et al., 2015; Yin et al., 2020). A negative surface charge is characteristic of almost all bacterial membranes and is caused by the corresponding high proportion of anionic phospholipids and associated amphiphiles on these membranes (Wang et al., 2013). Additional labeling methods include bacterial targeting probes; previous studies have used antibodies (Bispo et al., 2020), sugars (Geva-Zatorsky et al., 2015), lectins (Yang et al., 2012), antibiotic drugs (Stone et al., 2020), and peptides (Akram et al., 2015). However, other labeling methods involve the permeabilization of bacteria (Yang et al., 2013). Therefore, these methods are complex and may cause damage to the bacteria.

Commonly used fluorescent markers include fluorescent/bioluminescent proteins, fluorescent dyes, radioisotopes, and quantum dots (Peñate-Medina et al., 2019; Welling et al., 2019; Geng et al., 2020; Mota et al., 2020). Cyanine fluorescent dyes are of particular interest because of their favorable optical properties such as high molar extinction coefficients, narrow absorption/emission bands, satisfactory fluorescence quantum yields, and low toxicity to biosamples (Li et al., 2020). Consequently, these dyes have been widely used for the fluorescent labeling of eukaryotic cells and *in vivo* imaging of small animals (Selvam et al., 2011; Lacroix et al., 2019). In contrast to eukaryotes, bacterial cells consist of an outer membrane, a rigid peptidoglycan cell wall, a tight cytoplasmic membrane (Peabody et al., 2016), and a cellular structure that limits the endocytosis of cyanine 5.5 (Cy5.5).

**FIGURE 1:**
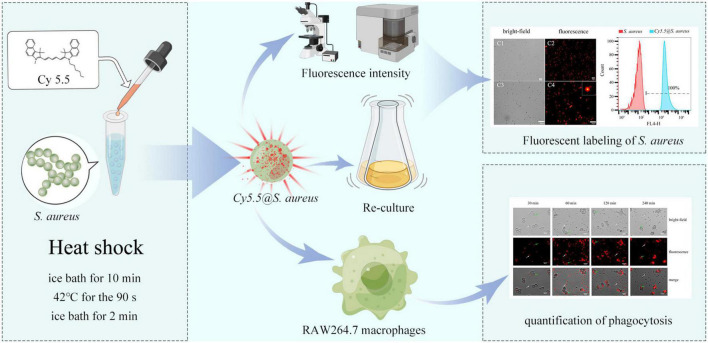
Schematic to represent the experimental process used in this study. Intracellular labeling of *Staphylococcus aureus* is achieved by heat shock with Cy5.5. The cytotoxicity of Cy5.5 and the stability of *Cy5.5@S. aureus* was also evaluated by flow cytometry. Additionally, *Cy5.5@S. aureus* was used to explore the phagocytic behavior of RAW264.7 macrophages.

Therefore, this study aimed to establish a simple and effective method for Cy5.5 labeling of *S. aureus* using a heat shock strategy. Then, fluorescently labeled *S. aureus* was used to detect the phagocytic behavior of RAW264.7 macrophages (Figure 1). Our labeled strains completely retained their biological activities and could be used to further study the interactions between host cells and invading bacteria; additionally, this method can also be applied to the real-time study of the dynamic processes of *in vivo* tracing following bacterial infection.

## 2. Materials and methods

### 2.1. Reagents and materials

Cy5.5, with an excitation/emission of 673/692 nm, was purchased from the DuoFluor Company. Enzyme-linked immunoassay (ELISA) kits for interleukin-6 (IL-6) and tumor necrosis factor-α (TNF-α) were obtained from Shanghai YanJin Biological. RPMI 1640 medium and fetal bovine serum (FBS) were purchased from Gibco. Nutrient broth (NB) and nutrient agar media were purchased from the Shanghai Comagal Microbial Company. *S. aureus* used in this study was preserved in our laboratory. The RAW264.7 macrophages were obtained from the National Key Laboratory of Medical Immunology and Institute of Immunology, Naval Medical University, Shanghai, China.

### 2.2. *Staphylococcus aureus* culture and cell culture

Single colonies of *S. aureus* were precultured in NB at 35°C overnight with shaking at 250 rpm, inoculated into fresh NB at 1:100 (v/v), and cultured at 37°C and 250 rpm (Coimbra et al., 2022) until an optical density of 0.6 at 600 nm (OD600) was attained. In all experiments, the bacteria were in the logarithmic growth phase.

RAW264.7 macrophages were cultured in RPMI 1640 medium supplemented with 10% FBS at 37°C and 5% CO_2_ in a humidified incubator (Yi et al., 2021). After 80% confluence of adherent cells, the culture flask was gently tapped and the cells were collected for experiments.

### 2.3. Bacterial labeling with Cy5.5

#### 2.3.1. Cy5.5 labeling of *Staphylococcus aureus* by heat shock

Bacteria in the logarithmic growth phase were washed three times with PBS (4,000 rpm, 10 min, 25°C), and the supernatant was discarded. The bacterial solution was then prepared with PBS at a concentration of approximately 2 × 10^8^ CFU/mL. Then, 2 μg/mL Cy5.5 was added to the bacterial solution; the solution was placed in an ice bath for 10 min, a water bath at 42°C for 90 s, immediately placed back into an ice bath for 2 min (Ma et al., 2021), then incubated for 60 min in a constant temperature and humidity incubator at 37°C. Finally, the sample was centrifuged at 4,000 rpm for 6 min, the supernatant was discarded and the pellet was resuspended in 1 mL of PBS.

#### 2.3.2. Flow cytometry

The fluorescence intensity of the labeled bacteria was measured using flow cytometry (BD Accuri C6 Plus); the FL4-H channel (excitation 640 nm, emission 675 nm) was used for fluorescence signal analysis and the positive region of the linear gate was set in the FL4-H histogram with unlabeled *S. aureus*. Positive events were used to express the labeling rate. Macrophage phagocytosis was quantified using flow cytometry by first setting the macrophage region according to the size and particle density in the forward scattered light (FSC) and side scattered light (SSC) two-dimensional scatter plots and setting the positive region in the FL4-H histogram with a linear gate using blank tubes (macrophages co-incubated with unlabeled bacteria). The positive rate was used to represent the phagocytosis rate. All measurements were performed with at least three independent replicates. All flow cytometric analyses were performed using FlowJo 10.8.

#### 2.3.3. Fluorescence imaging

*Cy5.5* near-infrared-I (NIR-I) dye–labeled *S. aureus (Cy5.5@S. aureus)* were applied to the slide, covered with a coverslip; fluorescence images were obtained with an inverted fluorescence microscope (Ming Mei MF-53-N) using a red filter with an excitation wavelength of 570–670 nm and an emission wavelength of 647–737 nm. Fluorescence images were taken at 20 × and 40 × magnification.

#### 2.3.4. Transmission electron microscope

Bacteria were washed three times with PBS, fixed with an electron microscope fixative at 25°C, and protected from light for 2 h. The samples were then fixed with 1% OsO_4_ for 2 h. After fixation, the samples were dehydrated with ethanol (from 30 to 100%), treated with acetone and epoxy resin for ultra-thin sections, and observed by transmission electron microscopy (TEM, HITACHI HT7700) (Shen et al., 2018).

#### 2.3.5. Cytotoxicity study of Cy5.5

Activated bacteria were incubated with 30 mL of fresh NB at 1:100 (v/v) with Cy5.5 dye added to give a final concentration of 2 μg/mL; alternatively, an unlabeled *S. aureus* control was produced. The bacterial suspension was incubated at 35°C with shaking at 250 rpm. A 150 μl sample was collected every 2 h, OD600 was measured using a BioPhotometer (Eppendorf), and growth curves were plotted.

#### 2.3.6. Re-culturing of *Cy5.5@S. aureus*

*Cy5.5@S. aureus* was inoculated with 30 mL of fresh NB at 1:50 (v/v) and incubated again at 35°C with shaking at 250 rpm; 1 mL of this culture was sampled every 2 h. The samples were washed three times with PBS, and the OD600 values were measured using a BioPhotometer. Fluorescence intensity was detected by flow cytometry, and fluorescence images were observed by fluorescence microscopy.

#### 2.3.7. Optimal conditions for Cy5.5 labeled *Staphylococcus aureus*

Cy5.5 dye at 0.5, 1, 2, 4, and 8 μg/mL was added to 1 mL of the bacterial solution (2 × 10^8^ CFU/mL). After the heat shock treatment, the cells were incubated at a constant temperature and humidity in an incubator at 37°C for 15, 30, 60, 120, or 180 min, respectively. After incubation, the fluorescence intensity and labeling rate were detected using flow cytometry, and fluorescence images were observed using a fluorescence microscope.

#### 2.3.8. Storage conditions for *Cy5.5@S. aureus*

*Cy5.5@S. aureu*s was stored at 25°C under natural light conditions or at 25, 4, or −20°C protected from light for 12, 24, 48, and 96 h. Samples were collected at each time point. In addition, *Cy5.5@S. aureus* was also sampled by freezing at −20°C followed by repeated thawing at 25°C five times. The mean fluorescence intensity (MFI) of the samples was examined using flow cytometry, and fluorescence images were observed using fluorescence microscopy.

### 2.4. RAW264.7 macrophages infection by *Cy5.5@S. aureus*

RAW264.7 macrophages in the exponential growth phase were inoculated in a 6-well plate at a concentration of 10^6^ cells/well and incubated at 37°C and 5% CO_2_ in a cell culture incubator for 4 h. After cells were plated, *Cy5.5@S. aureus* was added to each well at a multiplicity of infection of 20 (MOI = 20) and incubated for 30, 60, 120, and 240 min. At each time interval, the culture supernatant was collected, cells were washed three times with pre-chilled PBS, and fluorescence images were observed under a fluorescence microscope. Cells were fixed with paraformaldehyde at 25°C for 10 min, washed once with pre-chilled PBS, and the phagocytosis rate was detected by flow cytometry.

### 2.5. ELISA measures inflammatory factors in cell culture supernatants

The RAW264.7 macrophage culture supernatant was centrifuged at 4,000 rpm for 10 min to remove cell debris. The corresponding supernatant was used for cytokine quantification *via* ELISA. Specifically, the Levels of IL-6 and TNF-α were quantified by ELISA according to the manufacturer’s instructions. All measurements were performed with at least three independent biological replicates.

### 2.6. Statistical analysis

The experimental data were analyzed using SPSS software (version 26.0). Data are presented as mean ± standard deviation (SD). The significant differences between groups were compared using a Student’s *t*-test or ANOVA. A *P-*value < 0.05 was considered as statistically significant.

## 3. Results

### 3.1. Cy5.5 labeling of *Staphylococcus aureus* by heat shock method

**FIGURE 2:**
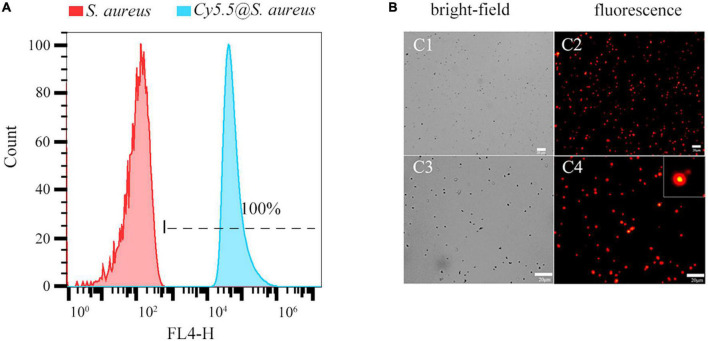
**(A)** Flow cytometry analysis of *Staphylococcus aureus* labeled with Cy5.5 by heat shock (FL4-H channel). Representative examples of unlabeled bacteria (red area) and *Cy5.5@S. aureus* (blue area) are shown. **(B)** Fluorescence micrographs of *S. aureus* after being labeled with Cy5.5 using a heat shock method, with *Cy5.5@S. aureus* shown in red (scale bar: 20 μm, the exposure time: 150 ms).

Fluorescence intensity was detected using the flow cytometry FL4-H channel. The labeling rate of *Cy5.5@S. aureus* reached 100% (Figure 2A), and its fluorescence intensity was significantly higher than that of the unlabeled *S. aureus* group (Figure 2A) and the *S. aureus* group that was directly incubated with Cy5.5 (Supplementary Figure 1). Fluorescence microscopy (Figure 2B) revealed that *Cy5.5@S. aureus* cells emitted bright red fluorescence intracellularly at an excitation wavelength of 680 nm. Overall, these results indicated that Cy5.5 penetrated *S. aureus via* heat shock treatment, thereby achieving successful labeling of *S. aureus*.

### 3.2. Optimal conditions for Cy5.5 labeled *Staphylococcus aureus*

**FIGURE 3:**
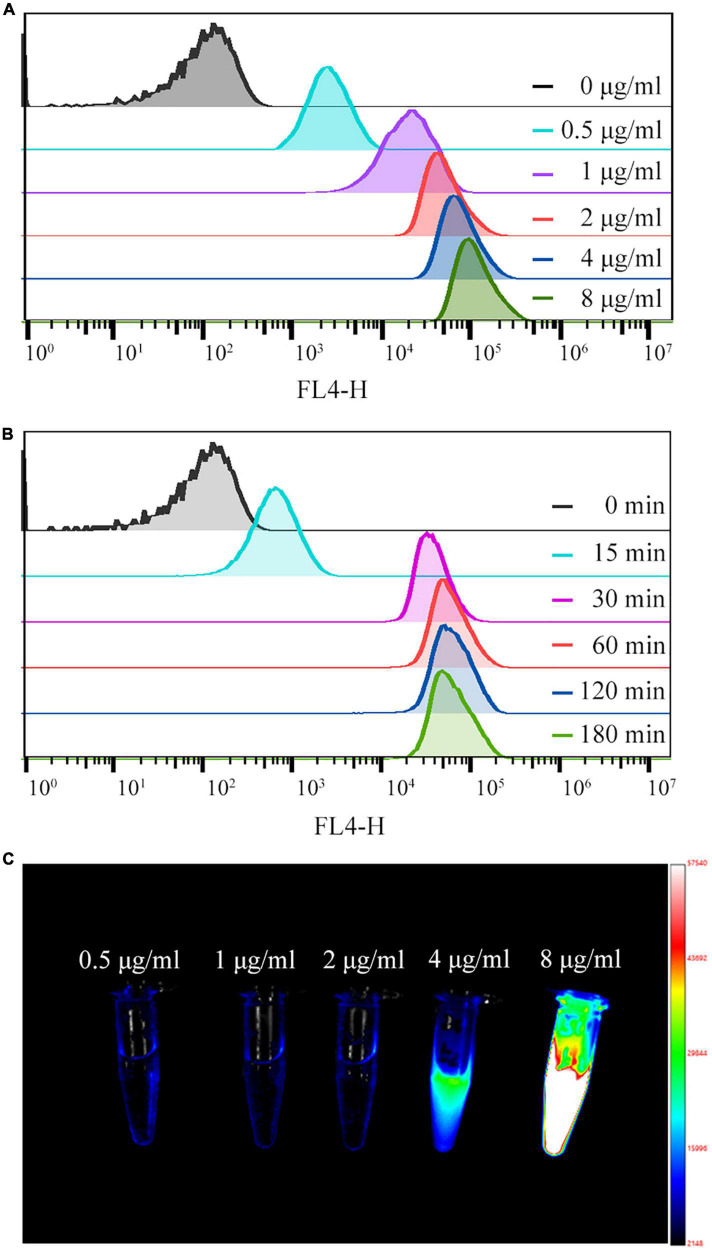
**(A,B)** Quantification of fluorescence intensity by flow cytometry for different concentrations of Cy5.5 dye (0.5–8 μg/mL) and for different labeling times (15–180 min). **(C)** NIR-I fluorescence image of the supernatant of *Staphylococcus aureus* after co-culture with different concentrations of Cy5.5.

The effect of Cy5.5 dye on the labeling rate and MFI of *S. aureus* was concentration-dependent over a range of concentrations. At Cy5.5 dye concentrations less than 2 μg/mL, the labeling rate and MFI increased rapidly with increasing dye concentration, reaching a 99.9% labeling rate at 1 μg/mL. Within the 2–8 μg/mL range, the MFI slowly increased with increasing Cy5.5 concentration (Figure 3A and Supplementary Table 1). To confirm these results, the original supernatant was collected. From the NIR-I images shown in Figure 3C, a strong fluorescence signal was detected in the supernatant at 4 and 8 μg/mL Cy5.5 concentrations, indicating a relatively high level of Cy5.5 compared to the *S. aureus* cells present.

We added 2 ug/mL of Cy5.5 dye to 1 mL of bacterial suspension (2 × 10^8^ CFU/mL). During labeling, MFI and labeling rate increased rapidly with time, reaching a 100% labeling rate at 30 min. The MFI was enhanced slowly within a labeling time of 30–60 min; however, after 60 min, the MFI did not change significantly with time (Figure 3B and Supplementary Table 2).

### 3.3. Storage conditions for *Cy5.5@S. aureus*

**FIGURE 4:**
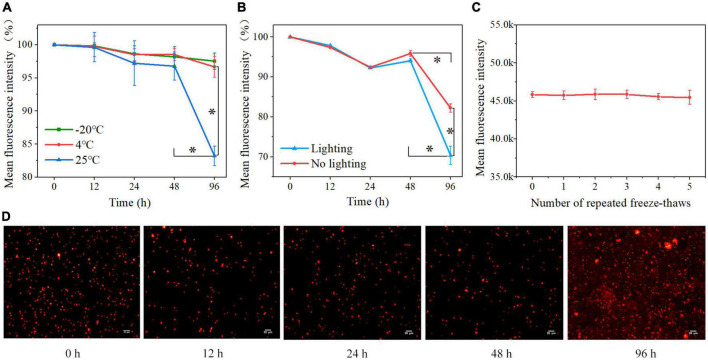
**(A)** Percentage loss of *Cy5.5@S. aureus* fluorescence signal over 96 h in light-protected storage at different temperatures **(B)** and under different light conditions at 25°C. The fluorescence intensity of *Cy5.5@S. aureus* stored for 0 h was 100%. **(C)** Effect of repeated freeze-thawing (5 times) on the fluorescence intensity of *Cy5.5@S. aureus*. **(D)** Fluorescence micrographs of *Cy5.5@S. aureus* at various time points during storage at 25°C for 96 h, with *Cy5.5@S. aureus* shown in red (scale bar: 20 μm, exposure time: 300 ms). Data are displayed as mean ± SD. *P < 0.01. *N* = 3 in each group.

*Cy5.5@S. aureus* was stored at 25, 4, and −20°C under light-protected conditions for various incubation times; the ratios of 12, 24, 48, and 96 h MFI to 0 h MFI were analyzed to evaluate the effect of storage temperature on the MFI of *Cy5.5@S. aureus*, using the MFI at 0 h as a reference (100%) (Figure 4A). Within 0–48 h, no significant change in MFI was observed after storage at 25, 4, and −20°C. At 48–96 h, there was no significant change in MFI at 4 and −20°C, but there was a significant decrease in MFI at 25°C (*P* < 0.01). At 96 h, the MFI decreased significantly at 25°C compared to that at 4 and −20°C (*P* < 0.01). These results indicate that the storage of *Cy5.5@S. aureus* at −20 and 4°C was superior to that at 25°C.

We also stored *Cy5.5@S. aureus* under natural light and light-protected conditions at 25°C, and analyzed the ratios of 12, 24, 48, and 96 h MFI to 0 h MFI to observe the effect of light on the MFI of *Cy5.5@S. aureus* (Figure 4B). From 0 to 48 h, the MFI did not change significantly under light-protected and natural light conditions. Within 48–96 h, the MFI decreased significantly (*P* < 0.01) in both light-protected and natural light storage; nonetheless, the MFI decreased more significantly (*P* < 0.01) in natural light storage than in light-sheltered storage. Fluorescence microscopy was also performed on *Cy5.5@S. aureus* stored under natural light at 25°C (Figure 4D). From 0 to 48 h, the fluorescence signals of the bacteria before and after storage were readable, and the background fluorescence signal of the bacteria was not significantly enhanced. At 96 h, the background fluorescence signal of bacteria was significantly enhanced. These results indicated that light significantly affected the fluorescence decay of *Cy5.5@S. aureus*.

The MFI of *Cy5.5@S. aureus* was not significantly changed after freezing at −20°C and repeated freeze-thaw at 25°C five times (Figure 4C), indicating that the fluorescence signal of *Cy5.5@S. aureus* was not significantly affected by repeated freeze-thawing.

### 3.4. Cytotoxicity assay of Cy5.5

**FIGURE 5:**
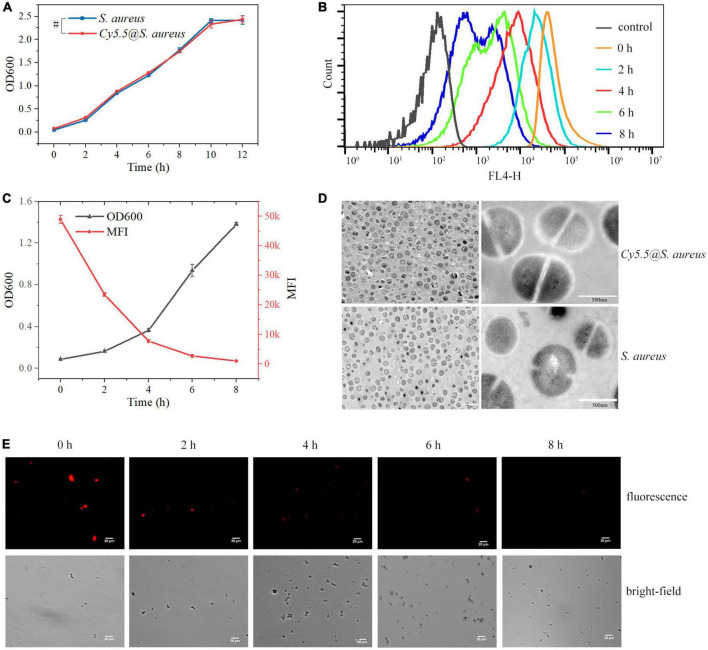
**(A)** Cytotoxicity assay of *Staphylococcus aureus* following incubation with Cy5.5 at a concentration of 2 μg/mL. Control refers to unlabeled *S. aureus*. **(B)** Quantitative analysis of fluorescence signal loss at various time points of *Cy5.5@S. aureus* re-culture by flow cytometry. Control refers to unlabeled *S. aureus* (FL4-H channel). **(C)** Double *Y-axis* plot of OD600 and fluorescence intensity of *Cy5.5@S. aureus* over incubation time. **(D)** TEM micrographs of *Cy5.5@S. aureus* and unlabeled *S. aureus*. **(E)** Fluorescence micrographs of *Cy5.5@S. aureus* cultured in NB at different time points, with *Cy5.5@S. aureus* shown in red (scale bar: 20 μm, exposure time: 300 ms). Data are displayed as mean ± SD. ^#^*P* > 0.05. *N* = 3 in each group.

Since there is a strong positive correlation between the turbidity of bacterial suspensions and the number of bacteria, bacterial cytotoxicity was measured by bacterial turbidimetry, as previously described (Perez-Soto et al., 2017). Activated *S. aureus* was incubated in 30 mL of fresh NB at 1:100 (v/v) with Cy 5.5 dye added at a final concentration of 2 μg/mL. Additionally, unlabeled *S. aureus* controls were used. The growth of bacteria in the experimental group with the addition of Cy5.5 did not differ significantly from that of the control group (*P* > 0.05) (Figure 5A). Therefore, it was surmised that Cy5.5 had no significant toxic effect on *S. aureus* at a dye concentration of 2 μg/mL.

### 3.5. Proliferation of *Cy5.5@S. aureus*

Next, we observed the proliferation of *Cy5.5@S. aureus*. The OD600 of *Cy5.5@S. aureus* cultures increased continuously with increasing incubation time (Figure 5C), indicating that our labeling method did not affect the proliferation of *Cy5.5@S. aureus*. In addition, the fluorescence intensity of *Cy5.5@S. aureus* decreased with the division and proliferation of the bacteria (Figures 5B, C). After 6 h of incubation, a clear fluorescence signal was no longer observed under a fluorescence microscope (Figure 5E). Therefore, it was inferred that the fluorescence intensity of *Cy5.5@S. aureus* decreased in a time-dependent manner, according to the cell division rate of *Cy5.5@S. aureus* in bacterial cultures. Therefore, the Cy5.5 dye in *Cy5.5@S. aureus* may be distributed to the daughter cells through bacterial division.

Further, cell morphology of *Cy5.5@S. aureus* was observed using TEM (Figure 5D), which showed that there was no significant morphological difference between *Cy5.5@S. aureus* and unlabeled *S. aureus*. It was concluded that labeling *S. aureus* using the heat shock method did not cause significant damage to the cells.

### 3.6. Quantification of the phagocytosis of *Cy5.5@S. aureus* in RAW264.7 macrophages

**FIGURE 6:**
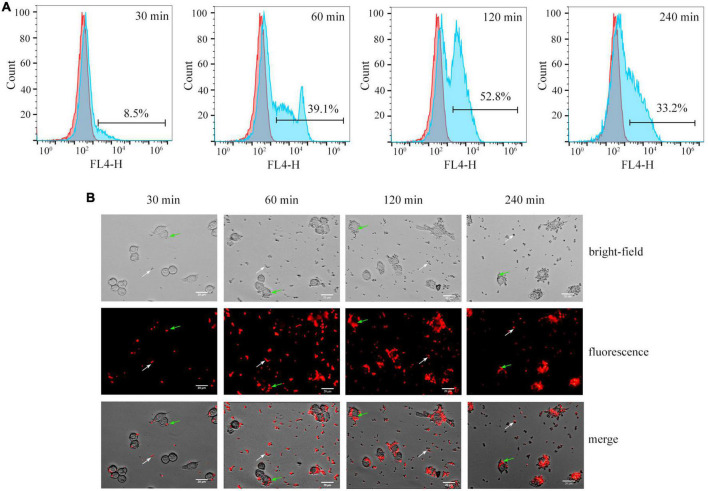
**(A)** Flow cytometry analysis showing the phagocytosis of *Cy5.5@S. aureus* by RAW264.7 macrophages at different times of co-culture *in vitro*. Representative examples of normal cells (red area), and cells that phagocytosed *Cy5.5@S. aureus* (blue area) are shown. **(B)** Fluorescence micrographs of *Cy5.5@S. aureus*–infected RAW264.7 cells at different time points. The white arrow indicates *Staphylococcus aureus* outside the macrophages and the green arrow indicates *S. aureus* that have been phagocytosed by the macrophages (scale bar: 20 μm, exposure time: 300 ms).

*Cy5.5@S. aureus* was co-cultured with RAW264.7 macrophages (MOI = 20) for 30, 60, 120, and 240 min. First, the phagocytosis of *Cy5.5@S. aureus* in RAW264.7 macrophages was detected using flow cytometry (Figure 6A). After 30 min of incubation, RAW264.7 macrophages gradually recognized and phagocytosed *Cy5.5@S. aureus* with a phagocytic rate of 8.5%. The phagocytosis rate of *Cy5.5@S. aureus* by RAW264.7 macrophages gradually increased over time; this rate reached 52.8% after 120 min of incubation but decreased to 33.2% after 240 min of incubation. This demonstrated that the maximum phagocytosis rate of macrophages was 52.8% at 120 min of infection, after which the phagocytosis rate decreased with prolonged infection time (*P* < 0.05) (Supplementary Figure 2). This decrease in phagocytosis rate may be attributable to the large number of dead cells with this prolonged infection time (Flannagan et al., 2016). Further, fluorescence microscopy revealed the phagocytosis of *Cy5.5@S. aureus* in RAW264.7 macrophages across these five time points (Figure 6B). At 30 min post-infection, *Cy5.5@S. aureus* were predominantly bound to the cellular membrane, as indicated by their corresponding red color. With the prolongation of co-culture time, RAW264.7 macrophages were gradually activated, and these cells showed a long shuttle, irregular shape. Bright red fluorescence was observed inside these macrophages. Analysis of intracellular MFI in macrophages showed a gradual increase with increasing infection time; a maximal intensity was observed at 120 min but the MFI at 240 min of infection was not statistically different from that at 120 min (*P* > 0.05) (Supplementary Figure 3).

**FIGURE 7:**
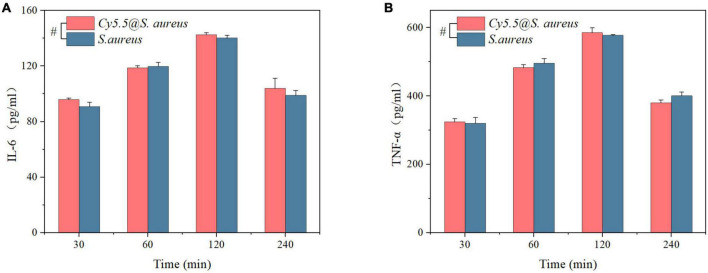
**(A,B)** ELISA analysis to detect the secretion of inflammatory factors (IL-6 and TNF-α) in the supernatant at different time points after infection of RAW264.7 macrophages with *Cy5.5@S. aureus* and unlabeled *Staphylococcus aureus*. Data are displayed as mean ± *SD.* ^#^*P* > 0.05. *N* = 3 in each group.

Additionally, the secretion of the pro-inflammatory factors TNF-α and IL-6 in cell culture supernatants at 30, 60, 120, and 240 min was examined. As shown in Figures 7A, B, *Cy5.5@S. aureus* stimulated RAW264.7 macrophages to secrete TNF-α and IL-6 with no significant differences compared to unlabeled *S. aureus* (*P* > 0.05). The secretion of TNF-α and IL-6 gradually increased with increasing co-culture time from 30 to 120 min. However, at 240 min, TNF-α and IL-6 secretion decreased. Overall, it was shown that *S. aureus* stimulated RAW264.7 macrophages to secrete TNF-α and IL-6, and *Cy5.5@S. aureus* had no significant effect on infectivity compared with unlabeled *S. aureus*.

## 4. Discussion

Cy5.5 is a commonly used NIR-I fluorescent dye that has been widely used in the fluorescent labeling of eukaryotic cells and *in vivo* imaging of small animals. Eukaryotic cells acquire macromolecules *via* endocytosis. Unlike eukaryotic cells, bacterial cells consist of an outer membrane, rigid peptidoglycan cell wall, and tight cytoplasmic membrane (Peabody et al., 2016); this cellular structure limits the endocytosis of Cy5.5. In this study, the intracellular labeling of *S. aureus* by Cy5.5 in was achieved by heat shock treatment, thereby providing a simple and effective method for the fluorescent labeling of bacteria that may be used for the quantification of bacterial invasion of eukaryotic cells and for *in vivo* tracing of bacterial infections.

Heat shock is a common method used for plasmid transformation (Kasarjian et al., 2003). Thermal excitation increases the temperature, releasing cell membrane lipids, forming pores in the cell membrane, and allowing DNA to enter the bacteria. After thermal excitation and cooling on ice for 2 min, the temperature decreases, the proteins of the cell membrane are released, the lipid percentage increases, the mobility of the cell membrane increases, and the pores in the cell membrane disappear (Panja et al., 2008). Using the heat shock method to label *S. aureus*, Cy5.5 entered the cytoplasm of the bacteria through the cell membrane pores and emitted bright red fluorescence inside these cells (Figure 2B). The labeling rate reached 100%, as determined by flow cytometry (Figure 2A). Nonetheless, a negative correlation was observed between bacterial proliferation and fluorescence intensity (Figures 5B, C). As these bacteria continued to proliferate, Cy5.5 was distributed to the daughter cells through the bacterial division. The labeling efficiency of *Cy5.5@S. aureus* was detected by flow cytometry for different dye concentrations and incubation times (Figures 3A, B). The combined analysis concluded that the optimal concentration range for Cy5.5 labeling of *S. aureus* (2 × 10^8^ CFU/mL) was 1–2 μg/mL and the optimal time for labeling was 30–60 min. The storage of bacteria after labeling had not been previously studied. In this study, *Cy5.5@S. aureus* was stored under different conditions, and the effects of storage temperature, light, and repeated freeze-thaw cycles on fluorescence intensity were analyzed (Figure 4). To facilitate the experiment and avoid bacterial wastage after labeling, we concluded that *Cy5.5@S. aureus* could be stored at 4°C for a short period.

The ideal bacterial labeling technique does not affect the proliferation and infectivity of bacteria. Aligning with this, we verified that Cy5.5 (2 μg/mL) had no significant toxic effect on *S. aureus* by comparing *S. aureus* co-cultured with Cy5.5 to unlabeled *S. aureus* (Figure 5A). *Cy5.5@S. aureus* was cultured in NB, and the fluorescence intensity of *Cy5.5@S. aureus* decreased as OD600 increased with increasing culture time (Figure 5B). This showed that our labeling method had no significant effect on the proliferation of *Cy5.5@S. aureus*. In addition, TEM analysis confirmed that labeling of *S. aureus* by the heat shock method did not significantly damage *S. aureus* (Figure 5D).

Macrophages play a critical role in fighting bacterial infections by phagocytosis, and possess a specific role in immune regulation by secreting pro-inflammatory cytokines, such as IL-6 and TNF-α (Hirayama et al., 2017). Therefore, we chose to coculture *Cy5.5@S. aureus* with RAW264.7 macrophages; the corresponding ELISA analysis for the pro-inflammatory factors TNF-α and IL-6 in the cell culture supernatants showed no significant difference between *Cy5.5@S. aureus* and unlabeled *S. aureus* (Figure 7). This suggests that our method had no significant adverse effects on *S. aureus* compared with unlabeled *S. aureus* infections. In contrast, Yang et al. (2013) adopted harsher methods for dye internalization in bacteria. In their study, the bacterial cell wall was severely disrupted, resulting in the leakage of the cell membrane. However, we intended to label our bacteria with minimal damage and, therefore, opted for a heat shock strategy.

Quantifying bacterial invasion of eukaryotic cells is a prerequisite for elucidating the molecular mechanisms of bacterial function. The invasiveness of *S. aureus* has traditionally been quantified by antibiotic protection assays, which require dilution plates and counting of clone-forming units rescued from infected cells after killing extracellular bacteria by preferential antibiotics (Pils et al., 2006). However, this assay may take up to 24 h to determine the results by colony count (Narayanan et al., 2013) and lacks reliability and reproducibility, as it tends to overestimate or underestimate internalized *S. aureus* (Agerer et al., 2004). Our labeled *Cy5.5@S. aureus* method allows real-time quantification of *S. aureus* invasiveness by flow cytometry and fluorescence microimaging, without the need to extracellularly kill the corresponding bacteria with antibiotics (Figure 6). Although some of the reported bacterial fluorescent labeling methods may be useful for research, including electrostatic adsorption (Yin et al., 2020) and antibodies (Bispo et al., 2020), these fluorescent staining methods require complex staining procedures, which may ultimately reduce bacterial viability. Alternatively, our labeling method is simple and does not impair bacterial viability. Therefore, real-time quantification of labeled *Cy5.5@S. aureus* using flow cytometry and fluorescence microimaging may be an alternative to traditional gentamicin protection assays. Moreover, *Cy5.5@S. aureus* may also be a possible method for *in vivo* tracing of bacterial infections owing to the excellent fluorescence properties of Cy5.5 dye. Nonetheless, this requires further experimental validation.

The ideal bacterial fluorescent marker *in vitro* can help to study the host–bacteria relationship; additionally, *in vivo* tracking of bacteria can help to understand the progress of the bacterial infection, which is important for the diagnosis and treatment of this infection (Drevets and Elliott, 1995). Our labeling method is simpler and does not impair the viability of bacteria, ultimately allowing real-time quantification of *S. aureus* invasiveness by flow cytometry and fluorescence microimaging. However, there were a few limitations to this method. First, unlike *S. aureus*, gram-negative bacteria have a complex cell envelope consisting of a plasma membrane, peptidoglycan cell wall, and outer membrane, with unique chemicals in the outer membrane, resulting in divergent physical properties. For example, when proteins diffuse freely in the plasma membrane, the movement of the outer membrane proteins is constrained in these gram-negative bacteria (Rojas et al., 2018). Further, owing to the presence of the outer membrane, labeling gram-negative bacteria by heat shock treatment is less efficient. Therefore, further optimization, such as chloroform-SDS or lysozyme-EDTA treatment, is needed for the labeling of gram-negative bacteria (Yang et al., 2013). Further, this method exhibits a gradually decrease in fluorescence signal as bacteria proliferate and does not enable a long imaging track period. Therefore, further experimental verification is required to determine how Cy5.5 dye release will interfere with the bacterial imaging tracking as the bacteria die.

In conclusion, we have achieved intracellular labeling of *S. aureus* by Cy5.5 using heat shock treatment and provided corresponding optimal labeling parameters and storage conditions. Additionally, the cytotoxicity of Cy5.5 and stability of *Cy5.5@S. aureus* were evaluated. Moreover, *Cy5.5@S. aureus* was used to quantify bacterial invasion into eukaryotic cells. The results demonstrated that heat shock is a simple and feasible method to obtain Cy5.5 fluorescently labeled bacteria with uniform fluorescence intensity and high luminance. More importantly, the properties of these bacterial surfaces were not altered with respect to cell-surface recognition and phagocytosis by macrophages.

## Data availability statement

The original contributions presented in this study are included in the article/Supplementary material, further inquiries can be directed to the corresponding author.

## Author contributions

FL performed experiments and wrote the manuscript. YJ helped perform the experiment. YZ and SC revised the manuscript. YT was responsible for the study. All authors contributed to the manuscript and approved the submitted version.
