# Supplementary material for: A simple and efficient fluorescent labeling method in Staphylococcus aureus for real-time tracking of invasive bacteria
Source: Front Microbiol. 2023 Feb 10;14:1128638. doi: 10.3389/fmicb.2023.1128638 (PMC9950555; doi:10.3389/fmicb.2023.1128638)
Supplement: Supplementary file 1 [file Data_Sheet_1.docx]

**Supplementary Table 1:** The efficiency of labeling *S. aureus* with different concentrations of Cy5.5

| Cy5.5 concentrations (μg/ml) | labeling rate (%) | MFI (10^3^) |
| --- | --- | --- |
| 0 | — | 0.1±0 |
| 0.2 | 92.3±0.5 | 1.6±0.2 |
| 0.5 | 98.7±0.6 | 3.2±0.6 |
| 1 | 99.9±0.6 | 24.6±6.9 |
| 2 | 100.0±0 | 52.0±5.8 |
| 4 | 100.0±0 | 73.6±5.1 |
| 8 | 100.0±0 | 99.2±6.9 |

Cy5.5 dye at 0.2, 0.5, 1, 2, 4, and 8 μg/ml was added to 1 ml of bacteria solution (2×10^8^ CFU/ml). After heat shock treatment, incubate in a constant temperature and humidity incubator at 37°C for 60 min. Analysis of MFI and labeling rate by flow cytometry. Data are mean ± SD. N=3 in each group.

**Supplementary Table 2:** The efficiency of 2 μg/ml Cy5.5 in labeling *S. aureus* at different labeling times

| labeling time (min) | labeling rate (%) | MFI (10^3^) |
| --- | --- | --- |
| 0 | — | 0.1±0 |
| 15 | 50.9±6.6 | 1.0±0.4 |
| 30 | 100.0±0 | 36.5±0.7 |
| 60 | 100.0±0 | 53.6±4.5 |
| 120 | 100.0±0 | 63.3±2.6 |
| 180 | 100.0±0 | 63.8±1.4 |

2 μg/ml of Cy5.5 dye was added to 1 ml of bacteria solution (2×10^8^ CFU/ml). After heat shock treatment, incubate in a constant temperature and humidity incubator at 37°C for 15, 30, 60, 120, and 180 min. Analysis of MFI and labeling rate by flow cytometry. Data are mean ± SD. N=3 in each group.


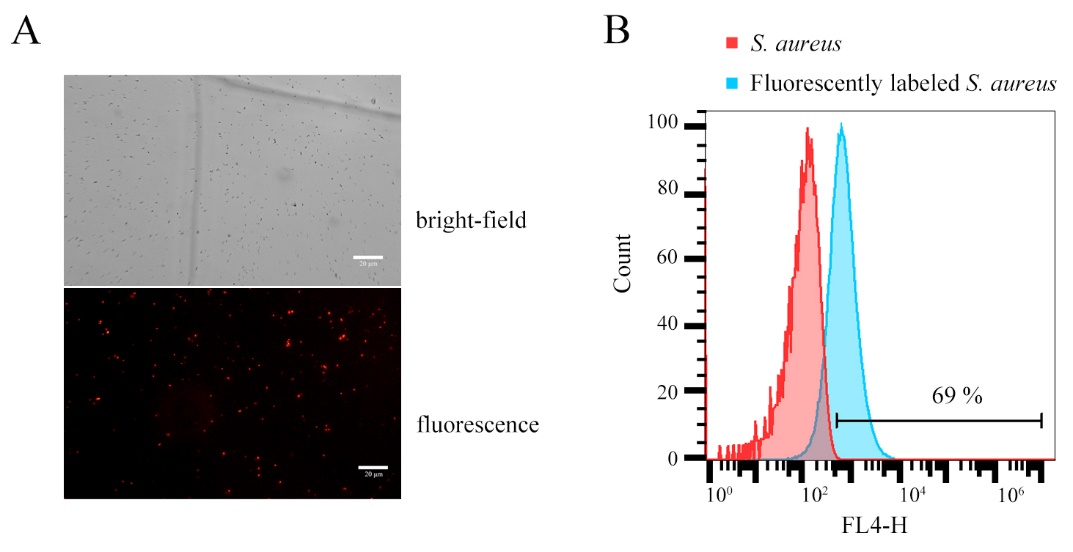


**Supplementary Figure 1:** (A) Fluorescence micrograph of *S. aureus* after direct incubation with Cy5.5, with Fluorescently labeled *S. aureus* in red (scale bar: 20 μm, the exposure time is 150 ms). (B) Flow cytometry analysis of *S. aureus* after direct incubation with Cy5.5 (FL4-H channel). Representative examples of unlabeled bacteria (red area) and Fluorescently labeled *S. aureus* (blue area) are displayed.


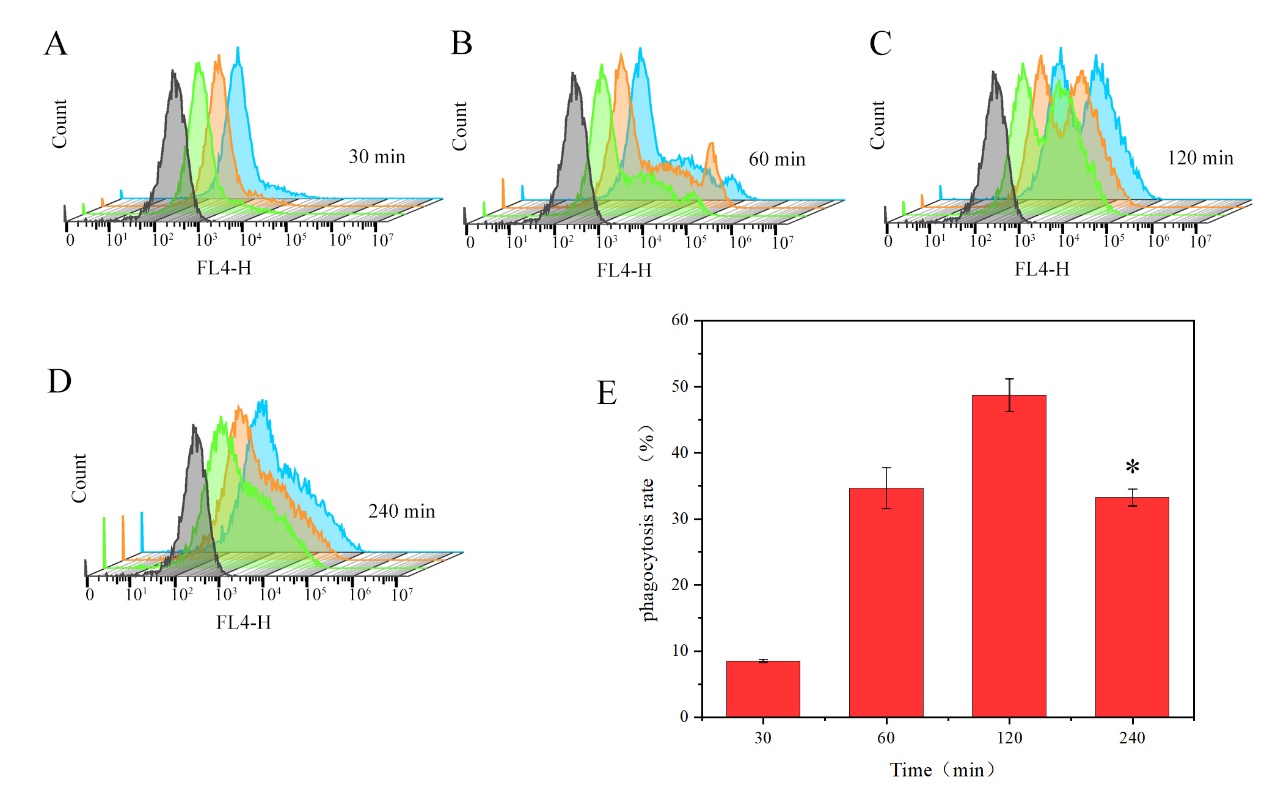


**Supplementary Figure 2:** (A-D) Flow cytometry determined the phagocytosis of *Cy5.5@S. aureus* by RAW264.7 macrophages at different times of co-culture in vitro. Representative examples of normal cells (black area), and cells that phagocytosed *Cy5.5@S. aureus* by repeating the experiment 3 times (colored areas) are displayed. (E) Histogram of phagocytosis of macrophages at 30, 60, 12, and 240 min of infection time. Data are mean ± SD. Compared to 120 min, ^*^*P*<0.05. N=3 in each group.


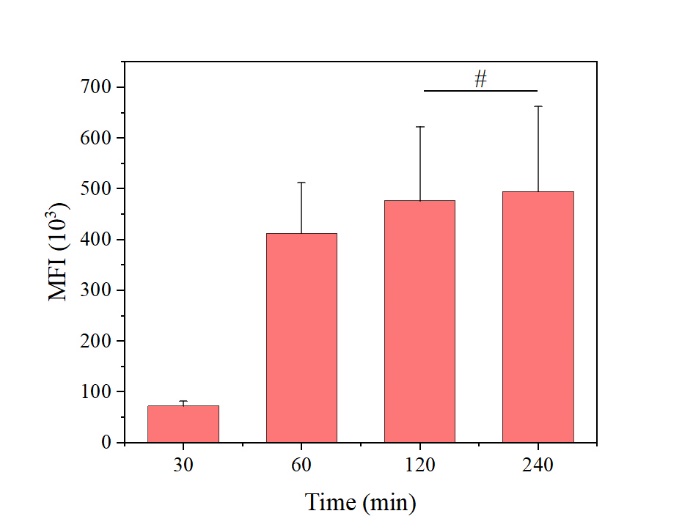


**Supplementary Figure 3:** Histogram of the mean fluorescence intensity in macrophages at 30, 60, 12, and 240 min of infection time. The data were analyzed by ImageJ software. Data are mean ± SD. ^#^*P*>0.05. N=3 in each group.
