# Supplementary figures and images for: A simple and efficient fluorescent labeling method in Staphylococcus aureus for real-time tracking of invasive bacteria
Source: Front Microbiol. 2023 Feb 10;14:1128638. doi: 10.3389/fmicb.2023.1128638 (PMC9950555; doi:10.3389/fmicb.2023.1128638)

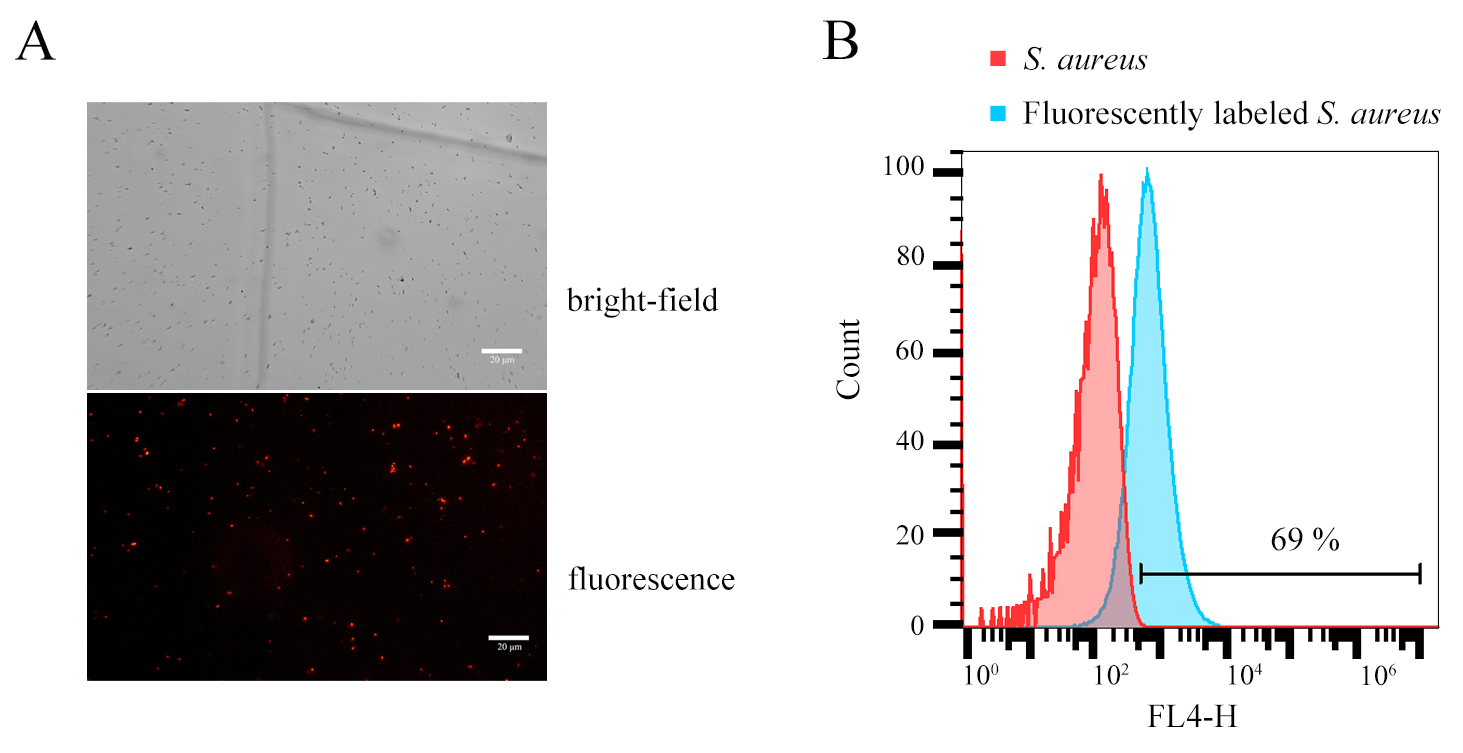

Supplement: Supplementary file 2 [file Image_1.JPEG]

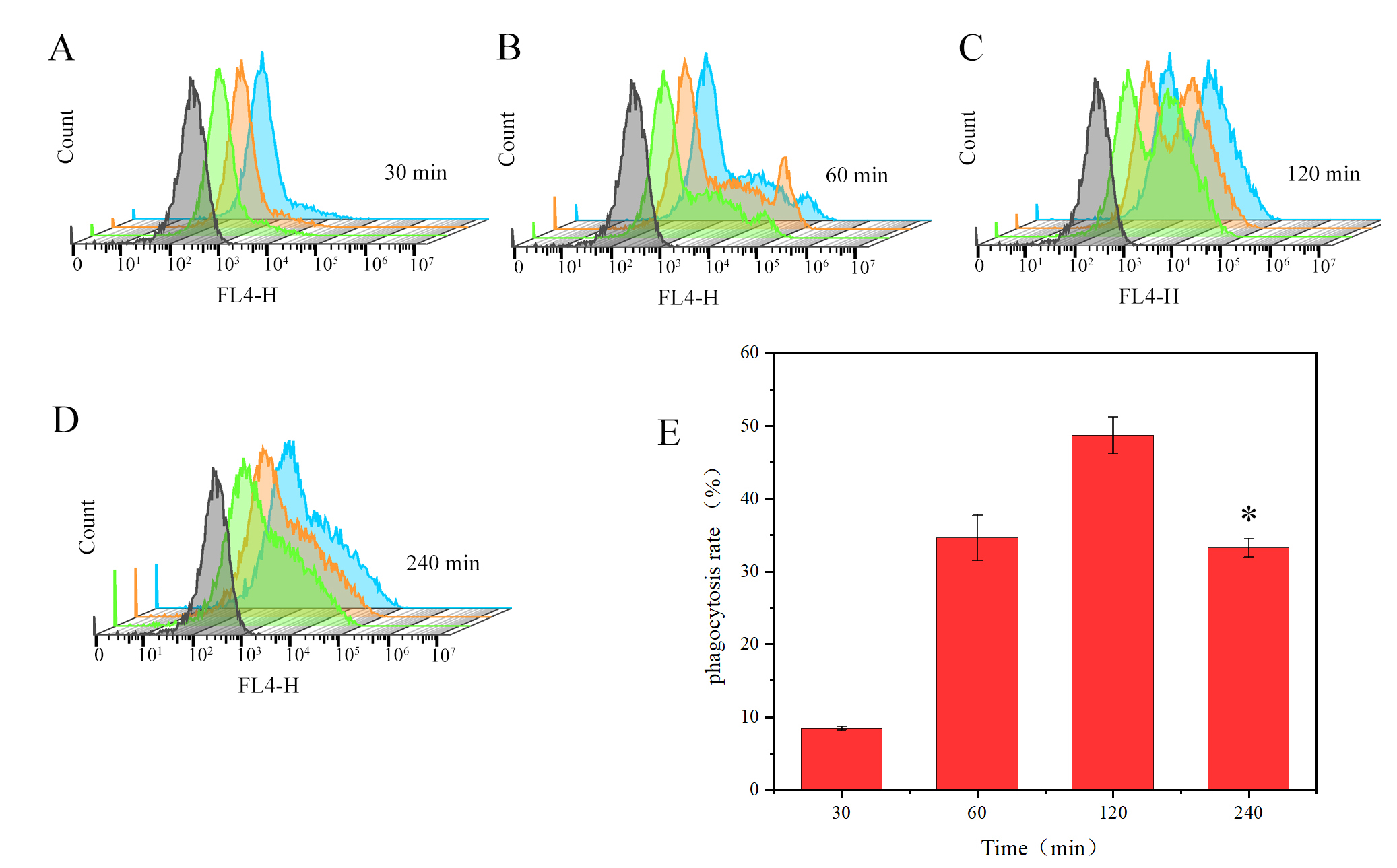

Supplement: Supplementary file 3 [file Image_2.JPEG]

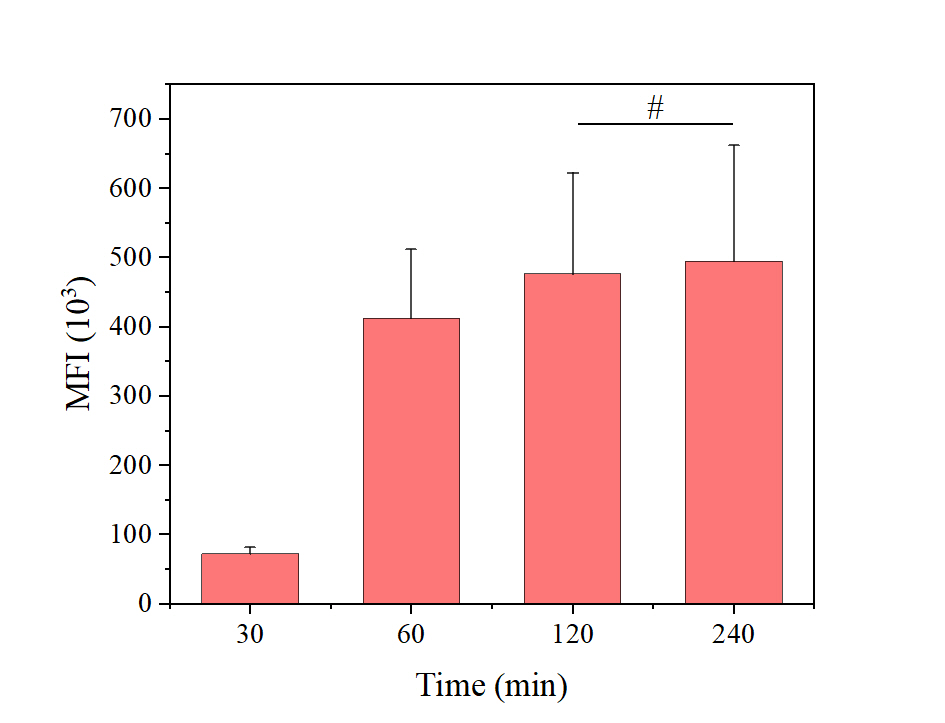

Supplement: Supplementary file 4 [file Image_3.JPEG]
